# Supplementary material for: Bacteroides fragilis polysaccharide A induces IL-10 secreting B and T cells that prevent viral encephalitis
Source: Nat Commun. 2019 May 14;10:2153. doi: 10.1038/s41467-019-09884-6 (PMC6517419; doi:10.1038/s41467-019-09884-6)
Supplement: Supplementary file 1 — Supplementary Information [file 41467_2019_9884_MOESM1_ESM.pdf]

Bacteroides fragilis Polysaccharide A Induces IL-10 Secreting B and T cells that Prevent Viral Encephalitis

Authors: Ramakrishna et al.

Supplementary Figures.

Supplementary Figure 1.

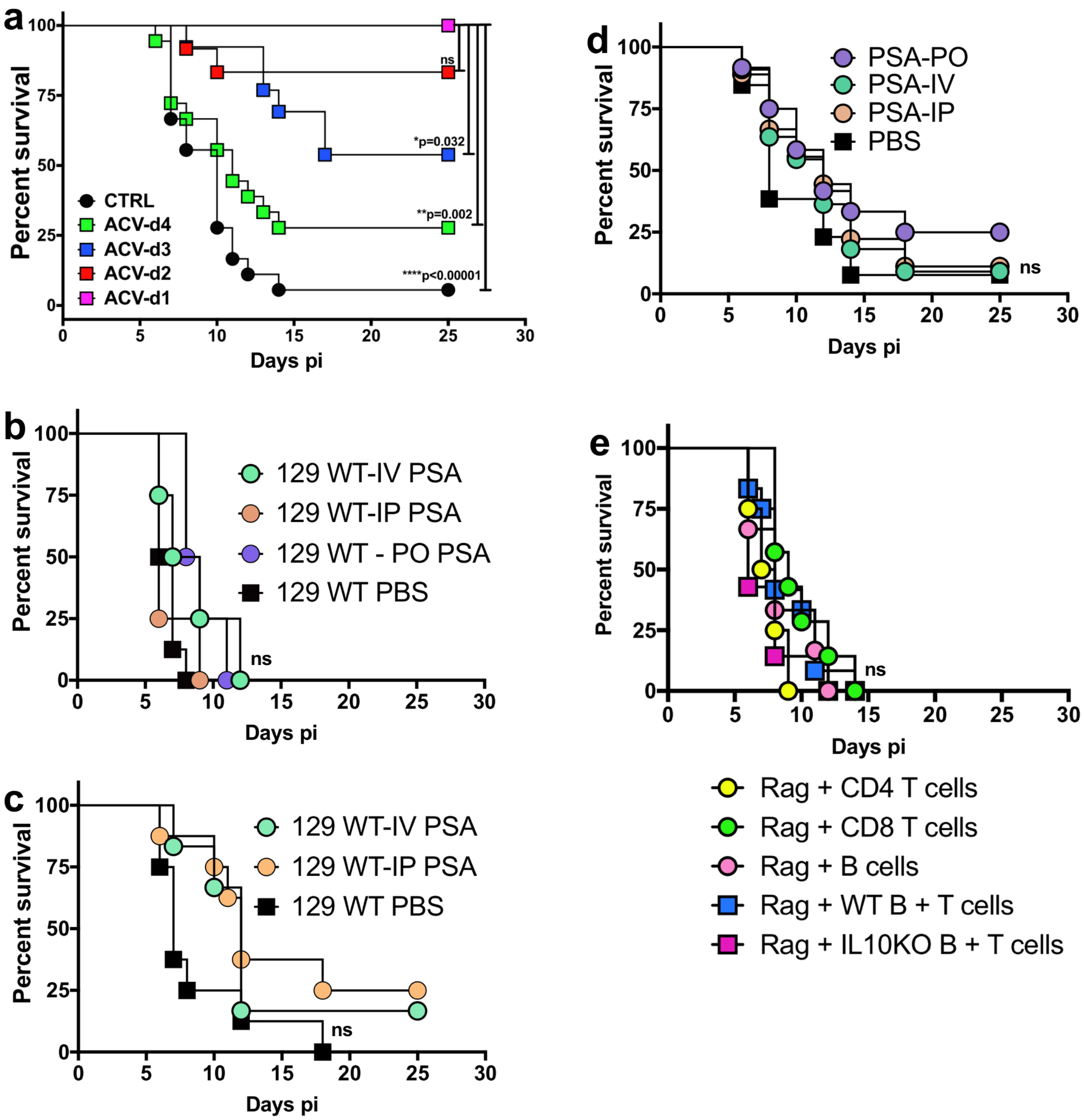

Supplementary Figure 1. Only prophylactic oral administration of PSA protects against HSE in a murine model with delayed ACV treatment.

(a) HSV infected 129 WT mice were given ip injections of ACV (1.25 mg/mouse) or PBS (CTRL) from the indicated time points for a week and monitored for HSE symptoms and survival (n=8-18 mice per group). HSV infected 129 WT mice given ACV from day 4 pi for a week by ip injections were also treated with PSA (50μg) or PBS by (**b)** ip, iv or oral (po) routes on days 1, 2 and 4 pi or (**c)** were pre-treated with 6 doses of PSA or PBS by ip or iv injections and monitored for survival, (n=6-8 mice per group, ns-not significant). (**d)** Another group of WT mice were pre-treated with PSA by the ip, iv or oral (po) routes but not given ACV after infection (n= 9-13 / group), ns=not significant. (**e)** PBS treated naïve Rag mice that were adoptively transferred with WT CD4 or CD8 T cells or WT B cells or WT B + T cells or IL-10KO B + T cells were infected with HSV and given daily ACV injections for a week starting on day 4 pi, (n= 6-12), ns= not significant. All statistical analysis was performed using log rank Mantel Cox survival test. See **Figures 1** and **5**.

Supplementary Figure 2.

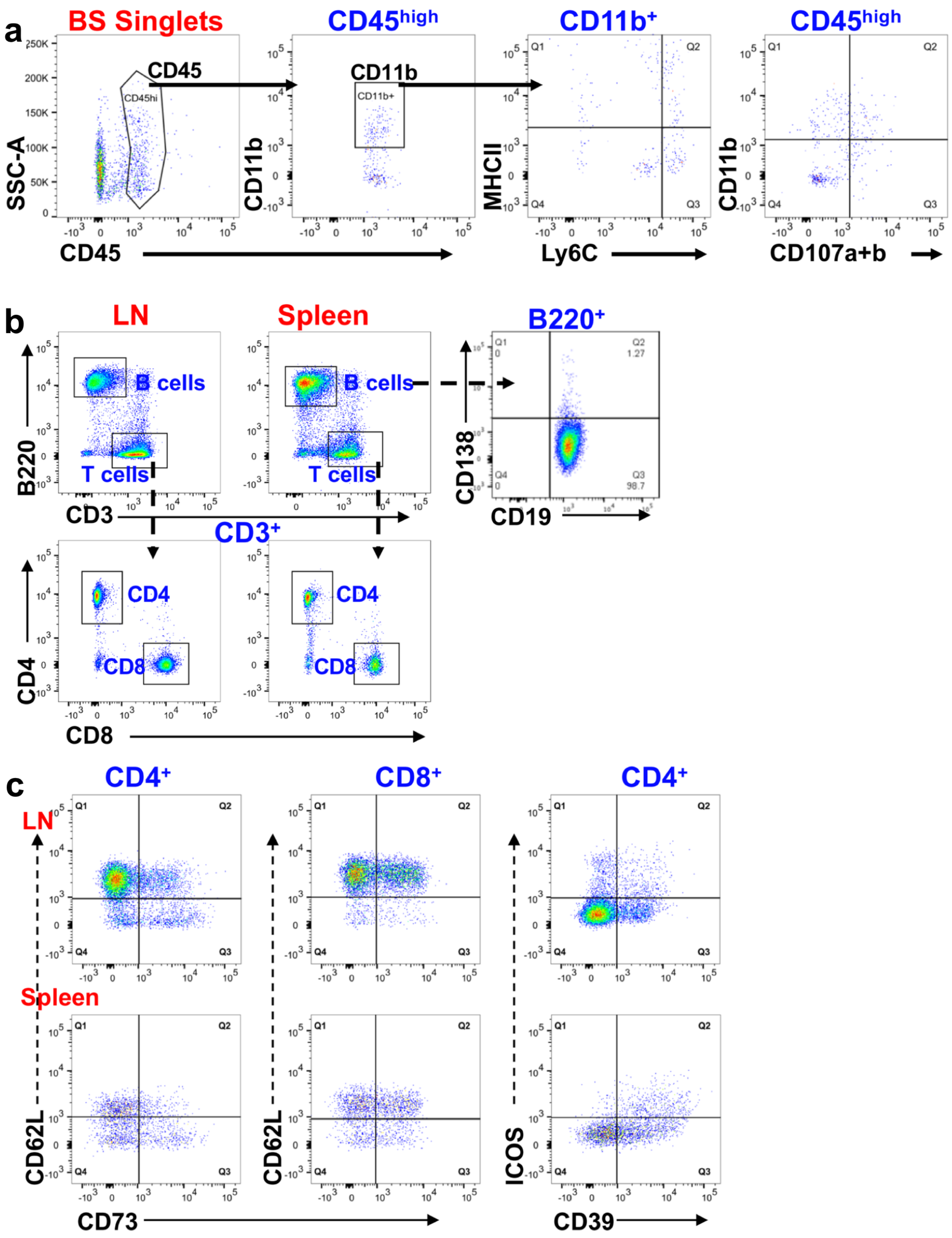

Supplementary Figure 2. Gating Strategy for analysis of cells in the brainstem (BS), lymph nodes and spleen.

(a) Mononuclear cells (live, singlets) isolated from the BS of infected mice at day 6 pi were analyzed for CD45 expression (left); $CD45^{high}$ gated cells were analyzed for $CD11b^{+}$ myeloid cells (left middle); $CD45^{high}CD11b^{+}$ gated cells were analyzed for MHC II and Ly6C expression to determine IM (right middle), PMN are MHC II^{-} $Ly6C^{int}$ $Ly6G^{+}$; $CD45^{high}$ gated cells were analyzed for degranulation of $CD11b^{+}$ cells by surface CD107a+b expression (right plot). This gating strategy was used to identify cells in the BS of WT and Rag mice and shown in **Figure 2, 4e-f, 5d-e** and **Supplementary Figures 5c**. (**b)** Mononuclear live singlet cells isolated from lymph nodes (LN: left plot) and spleen (middle plot) gated using lymphocyte gate were analyzed for B220 and CD3 expression, $B220^{+}$ gated cells were confirmed for CD19 expression (top right); $CD3^{+}$ gated T cells were analyzed for CD4 and CD8 expression (bottom panel). This gating strategy was used to identify and enumerate cells in LN and spleens of WT mice in **Figures 3, 4a-b, 6c-e** and **Supplementary Figures 2c, 3, 4c-d, 5a-b, 5d, 7, 8b-c and 1**0. **(c)** T cells from LN (top) and spleen (bottom) were gated on CD4 (left plot) or CD8 (middle plot) shown above in **(b)** and analyzed for CD73 and CD62L or ICOS and CD39 (right plot: CD4); See **Figures 2 and 3**.

Supplementary Figure 3.

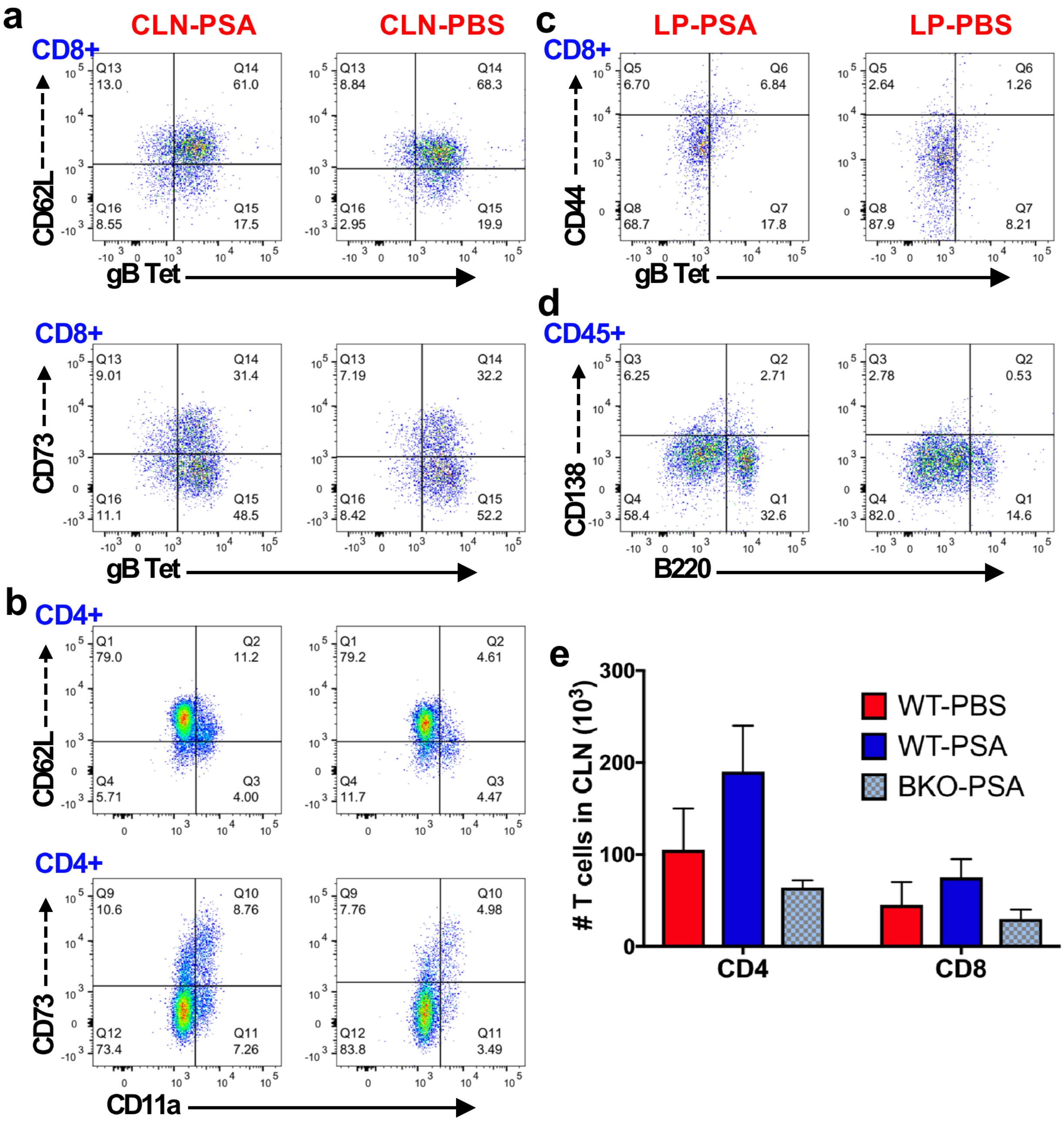

Supplementary Figure 3. PSA increases expansion of antigen specific T cells in lymphoid organs.

The draining cervical LN (CLN) isolated at day 6 pi from PSA or PBS pre-treated HSV infected, ACV treated WT mice were analyzed for accumulation of **(a)** CD8 T cells reactive to HSV $gB_{498-505}$ tetramer (gB Tet) and CD62L expression (top panel) or CD73 (bottom panel), **(b)** CD4 T cells expressing CD11a (as a surrogate marker for antigen experience) and CD62L (top panel) or CD73 (bottom panel). Naïve CD4 T cells do not express CD11a. **(c)** Mononuclear cells isolated from Lamina Propria (LP) at day 6 pi gated on $CD45^{+}$ $CD8^{+}$ T cells were analyzed for HSV $gB_{498-505}$ tetramer reactivity (gB Tet) and CD44 expression, and **(d)** $CD45^{+}$ cells were analyzed for plasma cells as determined by CD138 and $B220^{low}$ phenotype. **(e)** Bar plots shows total numbers of CD4 and CD8 T cells in the CLN of HSV infected 129 WT mice treated with PBS or PSA and 129 BKO mice treated with PSA (n=6 mice). All data show mean +/- SEM. (See **Figure 3 and 4**).

Supplementary Figure 4.

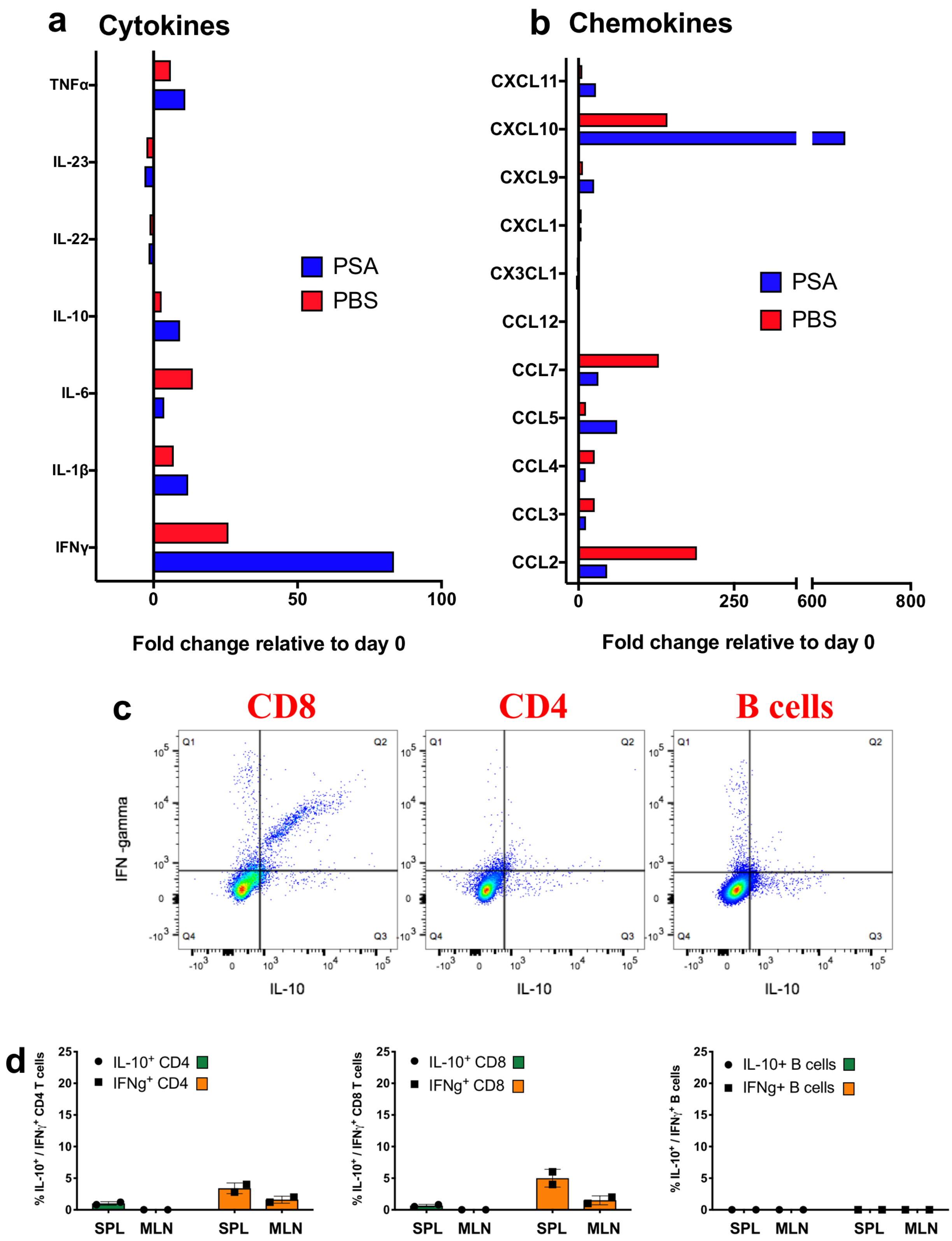

Supplementary Figure 4. Cytokine and Chemokine expression in the CNS and lymphoid organs.

(a) Cytokine and **(b)** chemokine expression in the BS of PSA (blue) or PBS (red) treated mice (n=3) at day 6 post HSV infection relative to uninfected mice (day 0); all mice were given ACV from day 4 pi. **(c)** Splenocytes were isolated at day 6 pi from PSA or PBS pre-treated HSV infected ACV treated WT mice and analyzed for intracellular IFNγ and IL-10 secretion following PMA and Ionomycin stimulation for 5 hours. Flow cytometry plots show CD8 (left) and CD4 (middle) T cell and B cell (right) gated cells. (**d)** Bar plots show CD4 (left) and CD8 T cells (middle) and B cells (right) isolated from spleens and MLN of WT mice treated with 6 doses of PSA orally that were analyzed prior to HSV infection for IL-10 and IFNγ secretion, n=2 experiments. All data show mean +/- SEM. See **Figure 4**.

Supplementary Figure 5.

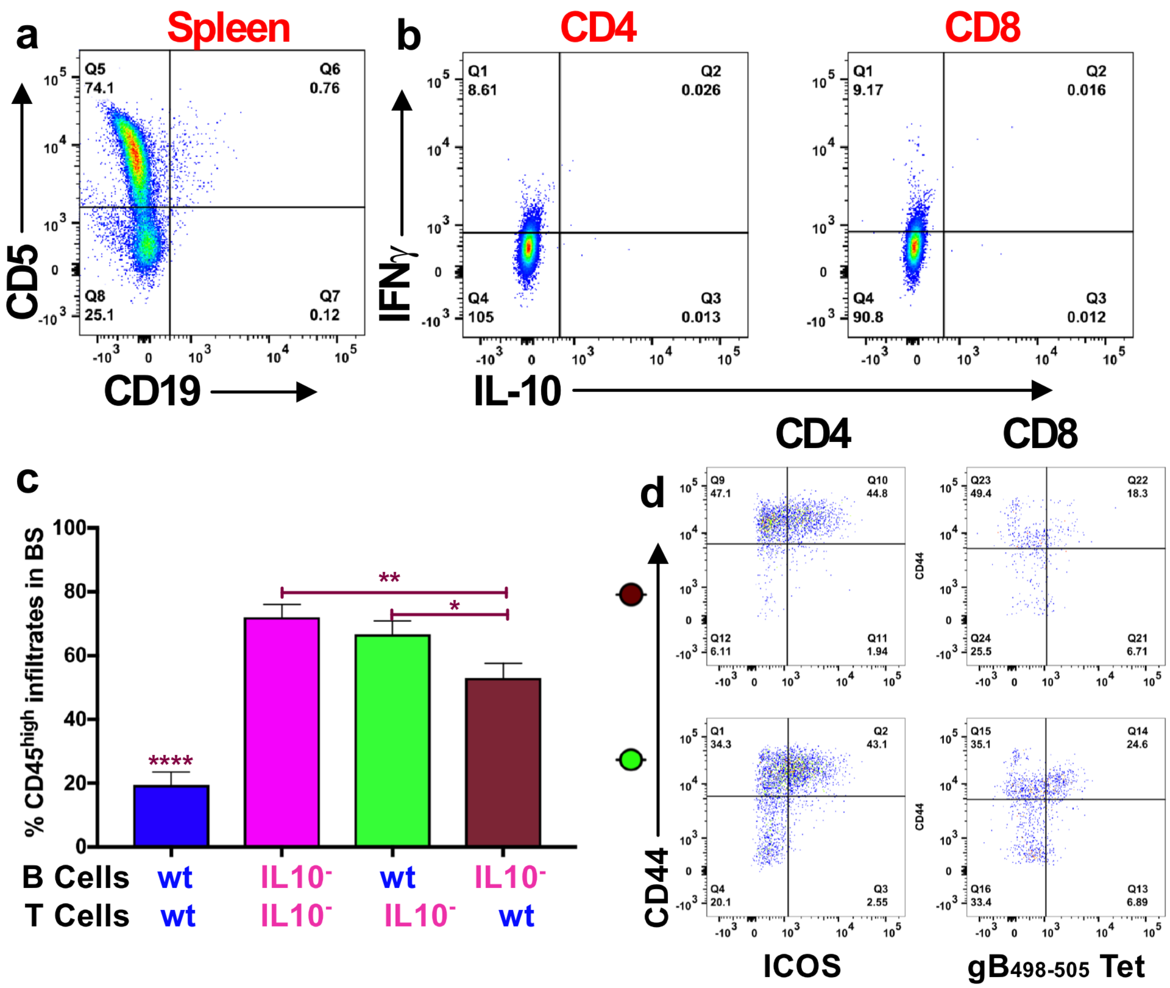

Supplementary Figure 5. PSA requires B cells to induce IL-10 secreting CD4 T cells.

(a) Flow cytometry plots show (left plot) complete depletion of $CD19^+$ B cells but not T cells ($CD5^+$) in the spleens of BKO mice at day 6 pi. To deplete B cells, WT mice were treated with αCD20 mAb given by ip injection every 10 days, beginning prior to PSA treatment and continued throughout infection (see also **Fig. 5b**). (**b)** Intracellular IFNγ and IL-10 expression by CD4 (middle plot) and CD8 (right plot) T cells isolated from spleens of BKO mice at day 6 pi following 5 hr stimulation with PMA + Ionomycin. (**c)** Bar plots show % $CD45^{high}$ cells infiltrating the BS of Rag recipients of WT B + T cells (blue), IL10KO B + T cells (magenta), WT B + IL10KO T cells (green) and IL10KO B + WT T cells (brown) at day 6 pi (see **Fig. 5d-e**), *p<0.05, **p<0.01, ****p<0.0001, as determined by ordinary one way ANOVA with Turkeys multiple comparisons tests. All data show mean +/- SEM. **(d)** Flow cytometry plots of spleens taken at day 6 pi from 129 Rag recipient mice of mixed transfers of IL-10KO B + WT T cells (top row: brown circle) and WT B + IL10KO T cells (bottom row: green circle) that were pre-treated with PSA prior to HSV infection show CD44 expression by (left plot) CD4 T cells and ICOS or (right plot) virus specific CD8 T cells reactive to HSV $gB_{498-505}$ tetramer (See **Figure 5d-e**).

Supplementary Figure 6.

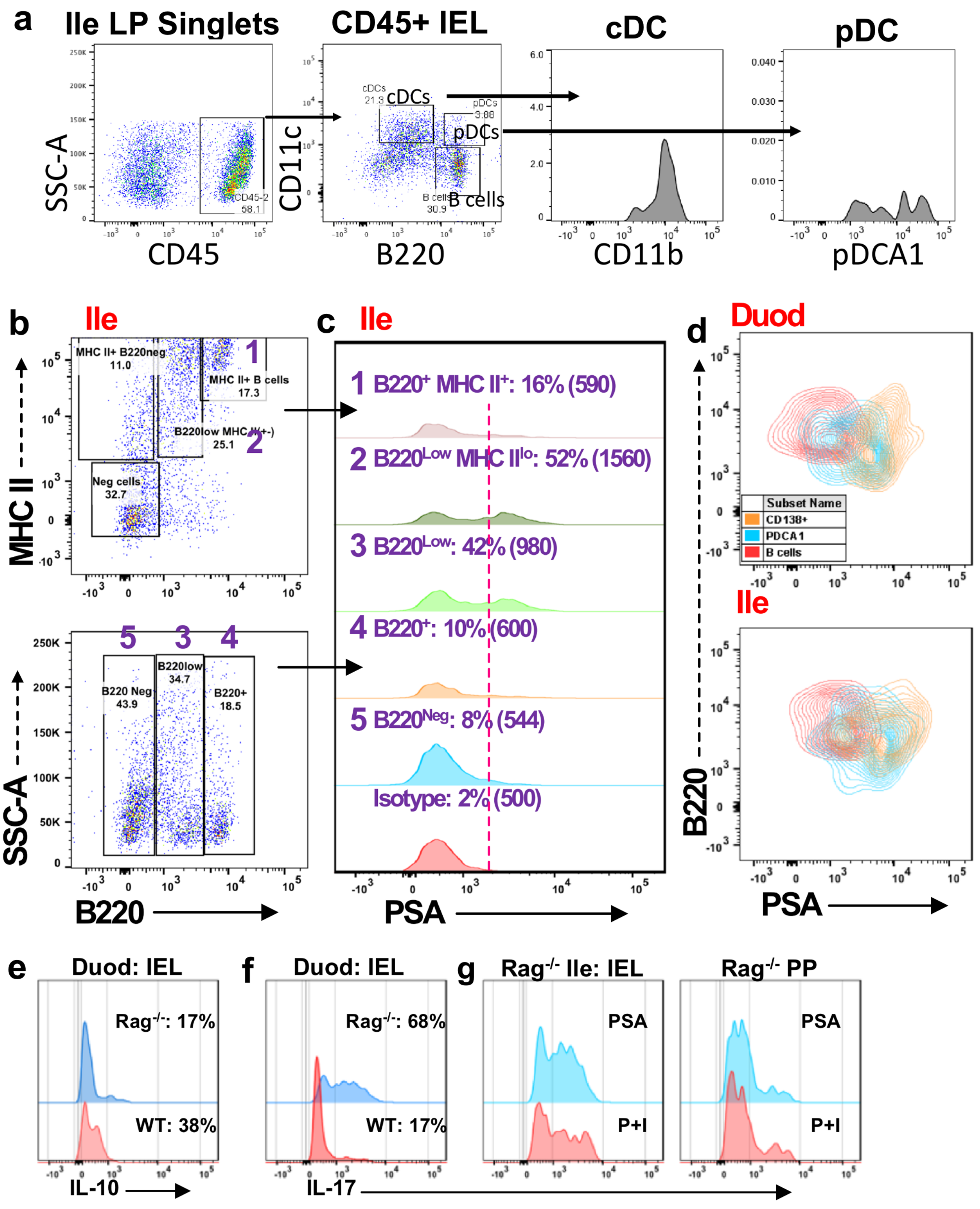

Supplementary Figure 6. PSA binding studies for gut resident B cells and IL-10 secretion by WT but not Rag gut cells.

(a) Gating strategy used to analyze Ileal $CD45^+$ mononuclear singlet IEL cells (left plot) to show CD45 $B220^+$ B cells, $B220^+$ $CD11c^+$ pDCs and $B220^-$ $CD11c^+$ cDCs (left middle plot). cDCs were confirmed by expression of CD11b (right middle histogram) while pDCs were confirmed by pDCA1 expression (right histogram). The gating strategy shown in **a** was used to identify immune cell types in the duodenum and ileum of WT and Rag mice as shown in **Figure 6** and **Supplementary Figures 6, 8a and 9b-c**. **(b)** Gating strategy used to analyze Ileal $CD45^+$ mononuclear singlet cells to show $B220^+$ cells with MHC II expression (top) and $B220^+$, $B220^{low}$ and $B220^-$ cells (bottom). **(c)** Gated populations represented by numbers 1-5 from the top and bottom plots in **(b)** were analyzed for binding to PSA, numbers show % PSA reactive while numbers in parenthesis show intensity of binding to PSA as denoted by MFI. **(d)** $CD45^+$ singlet mononuclear cells isolated from duodenum (Duod: top) and ileum (Ile: bottom) were gated on B cells, $CD138^+$ PCs and $PDCA1^+B220^+$ pDCs as shown in **(a)** and analyzed for binding to PSA. **(e)** $CD45^+$ IEL isolated from Duod of Rag (blue line) and WT (red line) mice (left 2 plots) showing IL-10 or **(f)** IL-17 secretion; **(g)** IL-17 secretion by Ile $CD45^+$ IEL (left) and PP cells (right) isolated from $Rag^{-/-}$ mice following stimulation with PSA (blue line) or PMA + Ionomycin (P+I: red line). See **Figure 6**.

Supplementary Figure 7.

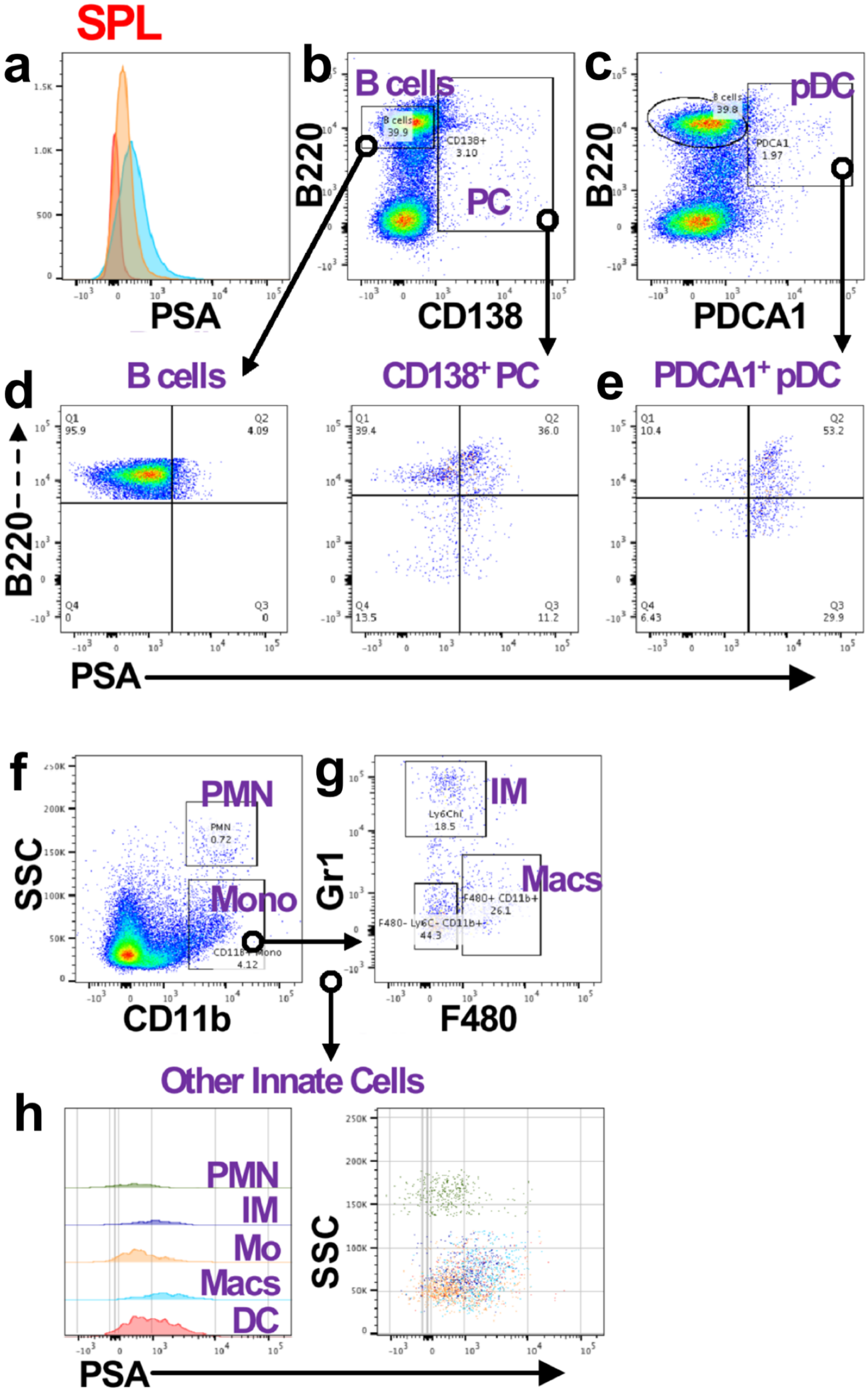

Supplementary Figure 7. PSA binding studies by spleen subsets.

(a), Top row, left histogram: Splenocytes from naïve WT mice were analyzed for binding to fluorescent conjugated PSA after pre-blocking with unlabeled PSA (yellow line) or PBS (blue line). Unlabeled cells: red line. Gating strategy to distinguish **(b)** $B220^{+}$ B cells and $CD138^{+}B220^{low}$ plasma cells (PC), **(c)** $B220^{+}PDCA1^{+}CD11c^{+}$ pDCs subsets (see arrows for primary gate) were analyzed for binding to PSA in **(d)** B cells (left) and PC (right), and **(e)** pDC. Gating strategy to analyze Innate cells including **(f)** $CD11b^{+}SSC^{high}Ly6G^{+}$ PMN and $SSC^{low}CD11b^{+}$ monocytes and **(g)** $SSC^{low}CD11b^{+}$ gated $F480^{+}CD11b^{+}$ macrophages (Macs) and $SSC^{low}Gr1^{high}F480^{-}$ IM (right plot). **(h)** Gated populations from plots **f and g** (see arrows for primary gate) were analyzed for PSA binding in **h**. This gating strategy was also used to identify spleen cell populations in **Supplementary Figure 8b-c**.

Supplementary Figure 8.

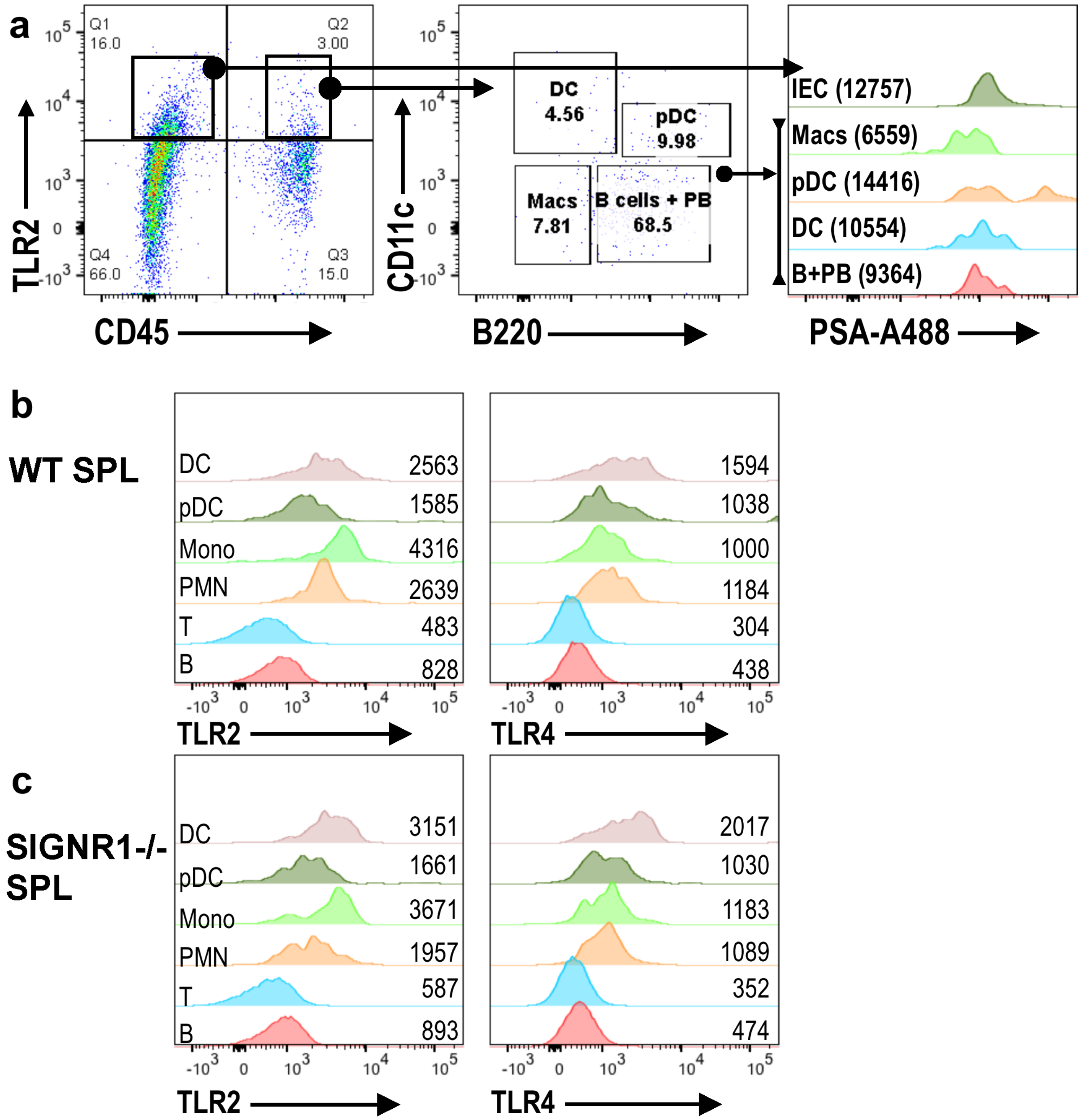

Supplementary Figure 8. TLR2 expressing cells bind PSA.

(a) Mononuclear live singlet cells isolated from duodenum of WT mice were analyzed for TLR2 and CD45 expression (left plot). $CD45^+$ $TLR2^+$ gated IEL cells were analyzed for CD11c, B220 and CD11b expression (middle plot); Gates show $CD11c^+B220^-$ cDCs, $B220^+CD11c^+$ pDCs, $B220^+CD11c^-CD19^+$ B cells + PB and $B220^-CD11c^-CD11b^+$ macrophages which were probed for binding to PSA (right plot), see **Supplementary Figure 6a** for gating strategy. $TLR2^+$ $CD45^-$ IEC (left plot) were also probed for binding to PSA in the right plot. **(b-c)** Various spleen cell subsets including B220- CD11c+ cDCs, PDCA1+CD11c+ pDCs, SSC^{low} CD11b+ monocytes/macs, SSC^{high} CD11b+ PMN, CD3+ T cells and B220+CD19+ B cells from **(b)** BALB/c WT and **(c)** SIGNR1KO mice were probed for TLR2 (left) and TLR4 (right) expression, see **Supplementary Figure 7** for gating strategy.

Supplementary Figure 9.

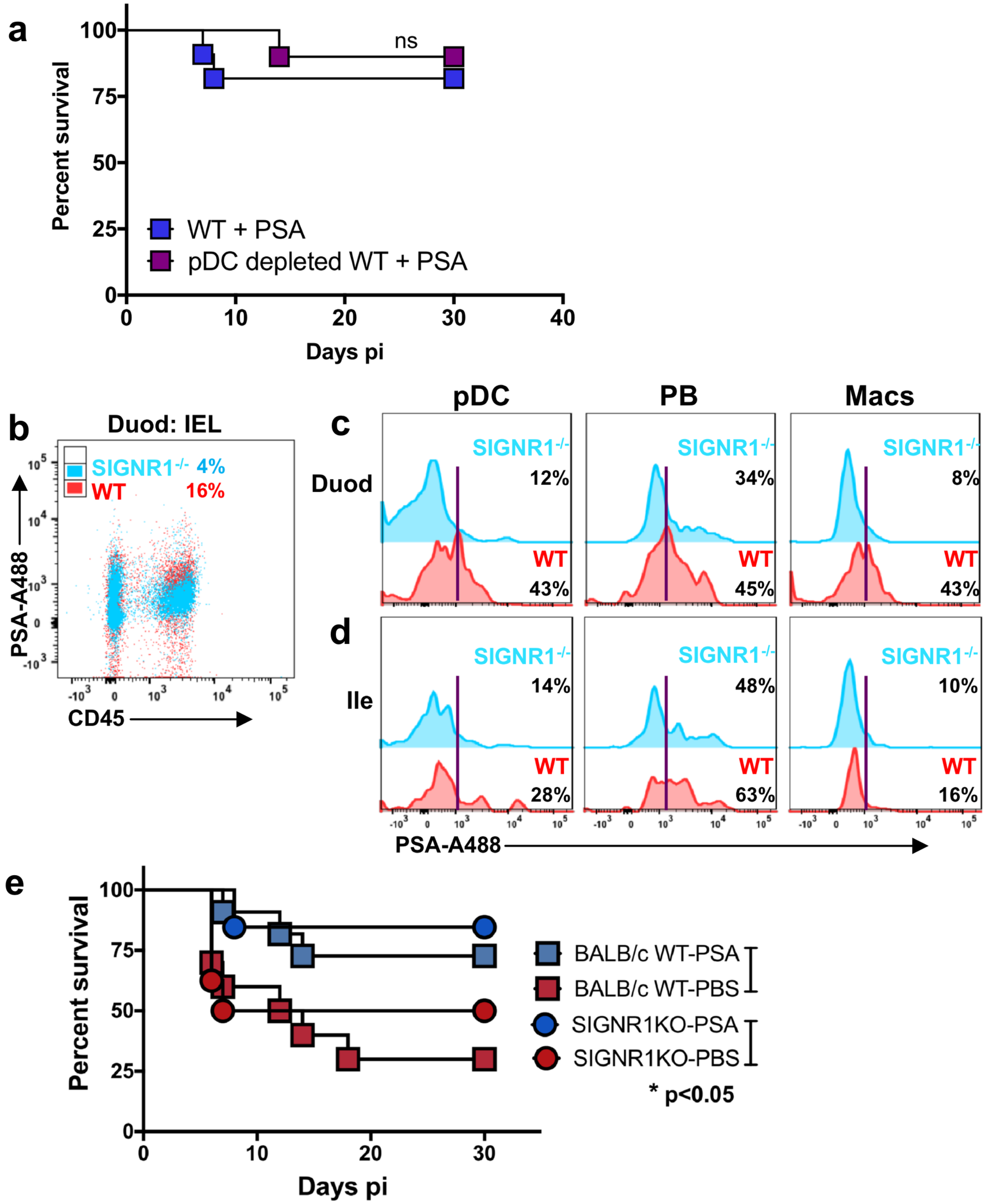

Supplementary Figure 9. Depletion of pDCs or SIGNR1 does not alter PSA mediated protection.

(a) WT mice were depleted of pDCs using anti-PDCA1 mAb prior to PSA pretreatment, and continued through HSV infection, mAb given every 3-4 days throughout experiment, n=10-12, ns=not significant. **(b)** IEL isolated from duodenum (Duod) of WT or SIGNR1$^{-/-}$ mice were analyzed for binding to PSA-A488. **(c-d)** pDCs (left), PB (middle) and F480^{+}CD11c^{-} macrophages (right) in the IELs isolated from **(c)** Duod and **(d)** Ile of WT (red line) and SIGNR1$^{-/-}$ (blue line) mice were analyzed for reactivity to PSA-A488. **(e)** SIGNR1KO or WT littermates were pre-treated with PSA or PBS as in Fig. 1a, infected with HSV 17+ (8x10^{4} PFU) by corneal scarification and treated with ACV from day 4 pi for one week; mice were monitored for symptoms of HSE and mortality (n=8-12), *$p<0.05$ as determined by log rank Mantel Cox test.

Supplementary Figure 10.

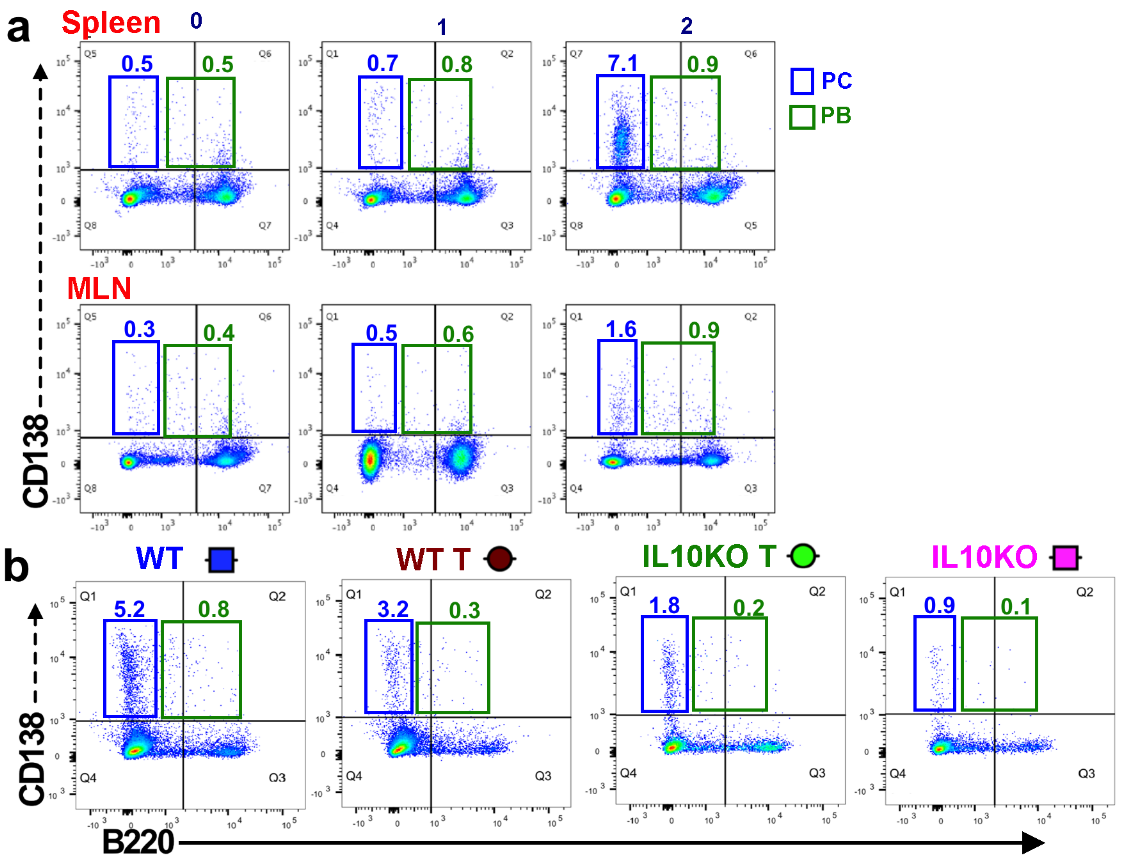

Supplementary Figure 10. T cell derived IL-10 essential for accumulation of plasma cells in the LN.

(a) Plasma cell ($CD138^{+}B220^{-}$ PC) and plasmablast ($CD138^{+}B220^{+}$ PB) accumulation in the spleens (top) and MLN (bottom) of WT mice prior to PSA treatment (left), after one week with 3 doses (1: middle) and after 2 weeks with 6 doses (2: right plot). Blue box labeled PC shows plasma cells and green box shows plasmablasts (PB). **(b)** Accumulation of PC and PB at day 6 pi in spleens of Rag recipients of WT B + T cells (WT: blue), IL10KO B + WT T cells (WT T: brown), WT B + IL10KO T cells (IL10KO T: green), and IL10KO B + T cells (IL10KO: magenta) that had been given 6 doses of PSA prior to HSV infection and ACV treatment. See **Figure 5** for key to symbols.
